# Titanium Dioxide Particle Type and Concentration Influence the Inflammatory Response in Caco-2 Cells

**DOI:** 10.3390/ijms17040576

**Published:** 2016-04-16

**Authors:** Saeko Tada-Oikawa, Gaku Ichihara, Hitomi Fukatsu, Yuka Shimanuki, Natsuki Tanaka, Eri Watanabe, Yuka Suzuki, Masahiko Murakami, Kiyora Izuoka, Jie Chang, Wenting Wu, Yoshiji Yamada, Sahoko Ichihara

**Affiliations:** 1 Department of Human Nutrition, School of Life Studies, Sugiyama Jogakuen University, Nagoya 464-8662, Japan; t-saeko@sugiyama-u.ac.jp (S.T.-O.); fha12aa100@st.sugiyama-u.ac.jp (H.F.); sya12aa057@st.sugiyama-u.ac.jp (Y.Sh.); tna12aa069@st.sugiyama-u.ac.jp (N.T.); 2 Graduate School of Regional Innovation Studies, Mie University, Tsu 514-8507, Japan; suzujohn@yahoo.co.jp (Y.Su.); 309111@m.mie-u.ac.jp (M.M.); izuoka@innov.mie-u.ac.jp (K.I.); jchang@suda.edu.cn (J.C.); wendywu1206@163.com (W.W.); 3 Department of Occupational and Environmental Health, Tokyo Univeristy of Science, Noda 278-8510, Japan; gak@rs.tus.ac.jp (G.I.); eri.watanabe.0503@gmail.com (E.W.); 4 Department of Human Genomics, Life Scinece Research Center, Mie University, Tsu 514-8507, Japan; yamada@gene.mie-u.ac.jp

**Keywords:** nanoparticles, titanium dioxide, food additive, intestinal epithelium, macrophage, inflammation, reactive oxygen species

## Abstract

Titanium dioxide (TiO_2_) nanoparticles are widely used in cosmetics, sunscreens, biomedicine, and food products. When used as a food additive, TiO_2_ nanoparticles are used in significant amounts as white food-coloring agents. However, the effects of TiO_2_ nanoparticles on the gastrointestinal tract remain unclear. The present study was designed to determine the effects of five TiO_2_ particles of different crystal structures and sizes in human epithelial colorectal adenocarcinoma (Caco-2) cells and THP-1 monocyte-derived macrophages. Twenty-four-hour exposure to anatase (primary particle size: 50 and 100 nm) and rutile (50 nm) TiO_2_ particles reduced cellular viability in a dose-dependent manner in THP-1 macrophages, but in not Caco-2 cells. However, 72-h exposure of Caco-2 cells to anatase (50 nm) TiO_2_ particles reduced cellular viability in a dose-dependent manner. The highest dose (50 µg/mL) of anatase (100 nm), rutile (50 nm), and P25 TiO_2_ particles also reduced cellular viability in Caco-2 cells. The production of reactive oxygen species tended to increase in both types of cells, irrespective of the type of TiO_2_ particle. Exposure of THP-1 macrophages to 50 µg/mL of anatase (50 nm) TiO_2_ particles increased interleukin (IL)-1β expression level, and exposure of Caco-2 cells to 50 µg/mL of anatase (50 nm) TiO_2_ particles also increased IL-8 expression. The results indicated that anatase TiO_2_ nanoparticles induced inflammatory responses compared with other TiO_2_ particles. Further studies are required to determine the *in vivo* relevance of these findings to avoid the hazards of ingested particles.

## 1. Introduction

Engineered nanoparticles (NPs), defined as particles with diameters of less than 100 nm, exhibit new physicochemical features at the nanoscale, such as large surface area, altered electronic properties, reactivity, and surface derivatization [1,2]. With the development of nanotechnology, an increasing number of nanoproducts are presently available in electronics, cosmetics, drug delivery systems, and food products [3,4]. Among them, titanium dioxide (TiO_2_) NPs are manufactured in large quantities and commercialized for various uses based on their high stability, photocatalytic effects, and whitening [5]. TiO_2_ NPs are typically synthesized in three different crystalline structures; anatase, rutile, or brookite [6], and the first two types are mainly used as industrial materials. Because of their low degree of toxicity and physicochemical properties, TiO_2_ NPs are widely used in a broad range of products, such as toothpaste, sunscreen, cosmetics, pharmaceuticals, and nanomedical reagents [7,8,9]. The use of engineered NPs in the food industry has been growing rapidly, based on added benefits, such as improved taste and texture, prolonged shelf life, and enhanced nutritional qualities [10].

Recent studies have demonstrated that TiO_2_ NPs are also used as food color additives and flavor enhancers [4]. Especially, high Ti amounts, e.g., 0.01–1 mg Ti per unit have been found in candies, sweets, and chewing-gums [11]. The study of Weir *et al.* [11] in the U.S. concluded that approximately 36% of TiO_2_ particles in food products were nanosized and indicated that the average exposure level to TiO_2_ particles was estimated at 1–2 mg TiO_2_/kg body weight (BW)/day for children and approximately 0.2–0.7 mg TiO_2_/kg BW/day for other consumers. Moreover, another study demonstrated that in six brands of sugar-coated chewing gum, >93% of TiO_2_ particles were smaller than 200 nm and 18%–44% of TiO_2_ particles were smaller than 100 nm, and around 95% of the nano-TiO_2_ particles were swallowed when a person chewed the gum [12]. While oral exposure to TiO_2_ NPs seems unavoidable, consumers have raised concern on the introduction of nanotechnology in the food without any safety information. Therefore, it is important to investigate the effects of TiO_2_ NPs on the gastrointestinal tract for safety assessment of TiO_2_ NPs. However, the majority of studies on TiO_2_ NPs toxicity investigated the risks associated with inhalation [13] and only a few data are available on the toxicity and cellular responses of intestinal cells to exposure to TiO_2_ NPs. The purpose of the present study was to determine the effects of exposure to TiO_2_ particles of different crystal structure and size in human epithelial colorectal adenocarcinoma (Caco-2) cells and THP-1 monocyte-derived macrophages.

## 2. Results

### 2.1. Characterization of Suspensions of Titanium Dioxide (TiO_2_) Particles

We selected 50 nm of anatase and rutile TiO_2_ particles as the nano-sized TiO_2_ particles and 100 nm of anatase and 250 nm of rutile TiO_2_ particles as the large-sized TiO_2_ particles. We also used TiO_2_ NPs (Degussa, P25; 21 nm), which are the standard materials in the field of photocatalytic reactions, contain anatase and rutile phases in a ratio of about 4:1 *w*/*w*.

**Figure 1:**
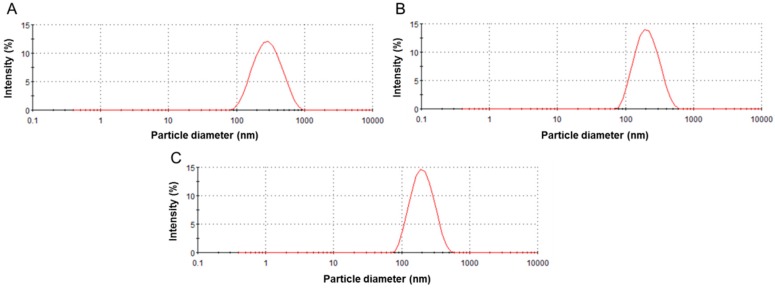
Histogram of particle size measured by dynamic light scattering technology. (**A**) anatase (50 nm); (**B**) rutile (50 nm); and (**C**) P25 (21 nm) titanium dioxide nanoparticles (TiO_2_ NPs) suspensions were dispersed using a sonicator (model 450, Branson Sonifier, Danbury, CT, USA) set at 100 watt (W), 80% pulse mode, for 15 min.

Nano-sized TiO_2_ particles were dispersed in the each culture medium of THP-1 and Caco-2 cells. The intensity-weighted hydrodynamic average diameter (*z*-average) of dispersed NPs was measured by the dynamic light scattering (DLS) technology, as described previously [14,15]. Table 1 shows the mean hydrodynamic diameters, polydispersity index (PdI), and ζ potential of dispersed TiO_2_ particles in each medium. The DLS data of TiO_2_ NPs indicated that the mean hydrodynamic diameter was >150 nm and confirmed the presence of nano-sized particles in the medium (Figure 1). The ζ potential of all particles in both cell media ranged from −11 to −14 mV (Table 1). No association between size or crystal structures and electrophoretic mobility of the particles was found.

### 2.2. Effects of Exposure on Cell Viability

THP-1 macrophages and Caco-2 cells were exposed to TiO_2_ particles at a concentration ranging from 1 to 50 µg/mL for 24 or 72 h.

**Figure 2:**
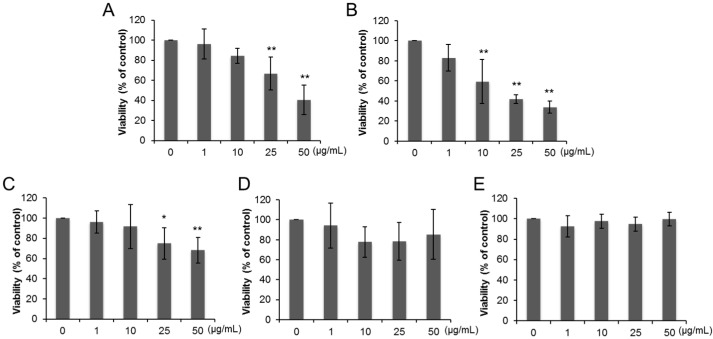
Cytotoxic effects of dispersed TiO_2_ particles on THP-1 macrophages. Cytotoxicity was measured by 3-(4,5-dimethylthiazol-2-yl)-5-(3-carboxymethoxyphenyl)-2-(4-sulfophenyl)-2H-tetrazo lium (MTS) assay (Promega, Madison, WI, USA). THP-1 macrophages were exposed to (**A**) A50; (**B**) A100; (**C**) R50; (**D**) R250; and (**E**) P25 at concentrations ranging from 1 to 50 µg/mL for 24 h. Data are mean ± SD of six experiments. * *p* < 0.05 *vs.* control (0 µg/mL). ** *p* < 0.01 *vs.* control (0 µg/mL).

**Figure 3:**
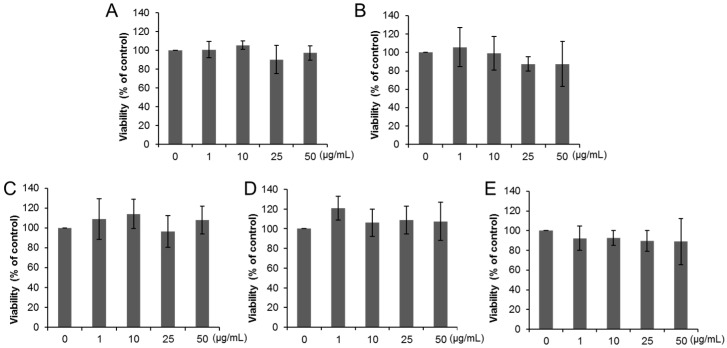
Cytotoxic effects of dispersed TiO_2_ particles on Caco-2 cells. Caco-2 cells were exposed to (**A**) A50; (**B**) A100; (**C**) R50; (**D**) R250; and (**E**) P25 at concentrations ranging from 1 to 50 µg/mL for 24 h. Data are mean ± SD of six experiments.

**Figure 4:**
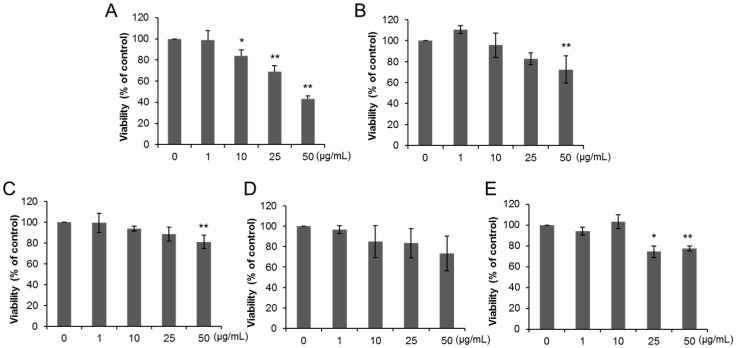
Cytotoxic effects of dispersed TiO_2_ particles on Caco-2 cells. Caco-2 cells were exposed to (**A**) A50; (**B**) A100; (**C**) R50; (**D**) R250; and (**E**) P25 at concentrations ranging from 1 to 50 µg/mL for 72 h. Data are mean ± SD of six experiments. * *p* < 0.05 *vs.* control (0 µg/mL). ** *p* < 0.01 *vs.* control (0 µg/mL).

Cell viability of THP-1 macrophages decreased after exposure to 25 and 50 µg/mL of anatase TiO_2_ particles of primary diameter of 50 nm (A50) (Figure 2A) and 10–50 µg/mL of anatase TiO_2_ particles of primary diameter of 100 nm (A100) (Figure 2B). Exposure to 25 and 50 µg/mL of rutile TiO_2_ particles of primary diameter of 50 nm (R50) also reduced the viability of THP-1 macrophages (Figure 2C), but no changes were noted in cells exposed to rutile TiO_2_ particles with primary diameter of 250 nm (R250) and P25 (Figure 2D,E). Figure 3 shows changes of viability of Caco-2 cells exposed to various TiO_2_ particles. No change in Caco-2 cell viability was evident following 24 h-exposure to all types of TiO_2_ particles (Figure 3A–E), while 72-h exposure to A50 (10, 25, 50 µg/mL, Figure 4A), A100 (50 µg/mL, Figure 4B), R50 (50 µg/mL, Figure 3C), and P25 (25, 50 µg/mL, Figure 4E) reduced their viability. However, no change was observed in R250-exposed cells (Figure 4D).

### 2.3. Effects of Exposure on Accumulation of Reactive Oxygen Species (ROS)

**Figure 5:**
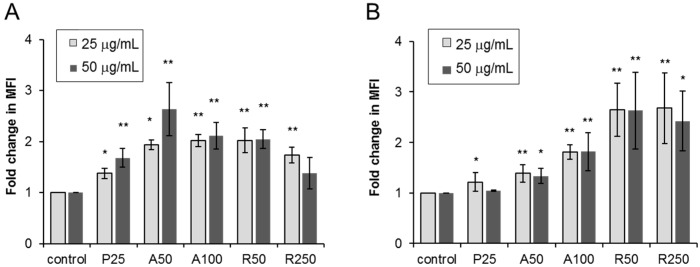
Effects of exposure of (**A**) THP-1 macrophages and (**B**) Caco-2 cells to dispersed TiO_2_ particles (concentration: 25 and 50 µg/mL, for 3 h) on ROS production. Data are mean ± SD of four experiments. * *p* < 0.05 *vs.* control (0 µg/mL). ** *p* < 0.01 *vs.* control (0 µg/mL).

ROS production occurs as the initial cellular response to foreign materials and the maximum ROS levels were found at 2–6-h exposure to TiO_2_ NPs by a previous *in vitro* study [16]. Therefore, we examined the effects of 3-h exposure of THP-1 macrophages and Caco-2 cells to TiO_2_ particles (25 and 50 µg/mL) on ROS production. Exposure to each type of TiO_2_ particles significantly increased ROS levels in THP-1 macrophages (Figure 5A), especially A50-exposed cells, and Caco-2 cells (Figure 5B).

### 2.4. Effects of Exposure on Interleukin (IL)-1β Levels in THP-1 Macrophages

**Figure 6:**
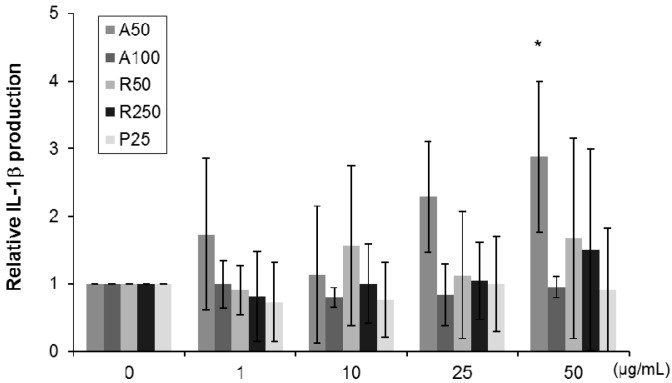
Effects of exposure of THP-1 macrophages to various TiO_2_ particles (concentration: 1–50 µg/mL for 24 h) on IL-1β production. Data are mean ± SD of three or four experiments. * *p* < 0.05 *vs.* control (0 µg/mL).

Increased production of inflammatory cytokine, IL-1β, was noted in THP-1 macrophages after 24-h exposure to A50, and such increase was dose-dependent, and the increase was significant at 50 µg/mL of A50 (Figure 6). However, exposure to other TiO_2_ particles had no significant effect on IL-1β level.

### 2.5. Effects of Exposure on Expression of IL-8 in Colorectal Adenocarcinoma (Caco-2) Cells

**Figure 7:**
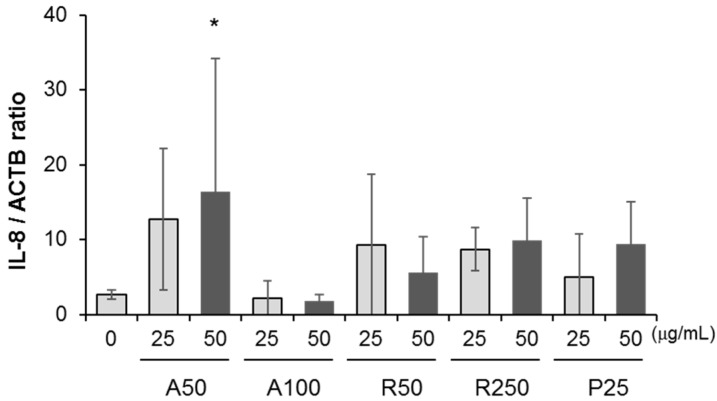
Effects of exposure of Caco-2 cells to various TiO_2_ particles (concentration: 25 and 50 µg/mL for 6 h) on IL-8 mRNA expression level. Data are mean ± SD of four experiments. * *p* < 0.05 *vs.* control (0 µg/mL).

We measured IL-8 expression in Caco-2 cells after 3- and 6-h exposure to particles because previous reports showed that inflammatory cytokine expression induced by NPs was detected after 1–6-h exposure [17,18]. There were no significant changes in IL-8 mRNA expression level in Caco-2 cells following 3-h exposure, irrespective of the type of TiO_2_ particle (25 or 50 µg/mL) (data not shown). However, exposure to 50 µg/mL of A50 for 6 h significantly increased IL-8 mRNA expression in Caco-2 cells (Figure 7).

## 3. Discussion

In the present study, we examined the effects of exposure to different crystal structures and sizes of TiO_2_ particles in Caco-2 cells and THP-1 monocyte-derived macrophages. Our results indicated that anatase TiO_2_ NPs induced inflammatory responses compared with other TiO_2_ particles.

Although the primary particle size of TiO_2_ particles is around 200–300 nm, smaller TiO_2_ NPs measuring 1–50 nm are currently used for the purpose of ultraviolet (UV) protection and photocatalytic activity. Previous studies examined the effects of inhalation exposure on inflammation and showed that ultrafine particles induced stronger inflammatory responses than fine particles [19]. On the other hand, pulmonary instillation studied showed that nanoscale particle types of TiO_2_ were not more cytotoxic or inflammogenic to the lung compared with larger sized particles of similar composition [20]. Other studies showed that inhalation of rutile ultrafine-TiO_2_ particles was less likely to be associated with adverse pulmonary health effects compared with anatase ultrafine-TiO_2_ particles [21]. Sayer *et al.* [22] showed that anatase TiO_2_ particles were 100 times more toxic than an equivalent sample of rutile TiO_2_ particles in human dermal fibroblasts and human lung epithelial cells. However, these studies focused mainly on the pulmonary toxicity of TiO_2_ NPs following intratracheal instillation and inhalation. Since TiO_2_ NPs have also been used recently as a white pigment and as a food additive for food coloring, determination of the effects of TiO_2_ NPs on the intestine is urgently needed for safety assessment of these particles.

The present study investigated the effects of exposure to different sizes of anatase and rutile TiO_2_ particles. The MTS assay showed that incubation of THP-1 macrophages in the presence of anatase TiO_2_ particles significantly reduced cell viability compared with rutile TiO_2_ particles. Moreover, incubation of Caco-2 cells in the presence of anatase TiO_2_ particles, especially anatase TiO_2_ NPs of primary particle size of 50 nm, significantly reduced cell viability after exposure for 72 h. We have recently reported the cytotoxicity of zinc oxide (ZnO) NPs, but not P25 TiO_2_ NPs, on endothelial cells [23]. The typical crystalline composition of P25 TiO_2_ NPs was around 80% anatase and 20% rutile [24]. The present study also showed that 24-h-exposure to P25 TiO_2_ NPs was not cytotoxic for both THP-1 macrophages and Caco-2 cells. The results indicate that anatase TiO_2_ NPs is more toxic than rutile TiO_2_ particles, suggesting that TiO_2_ particle toxicity in human intestinal cells depends on the particle size and crystalline structure.

Previous acute oral toxicity studies showed that TiO_2_ NPs had very low toxicity in animals [25,26]. Moreover, oral administration of TiO_2_ NPs showed low absorption and narrow range of organ distribution [27], but slow tissue elimination [28]. Although data using cultured cells are not a substitute for whole animal studies, the use of simple cell culture models with endpoints that can identify the mechanism of cellular responses or toxicity can be the basis for further assessment of the potential risk of material exposure. Previous cell culture studies showed that TiO_2_ NPs induced oxidative stress and increased IL-1β levels in murine dendritic cells [29]. Yazdi *et al.* [30] showed that TiO_2_ NPs activated the NLR pyrin domain containing 3 (Nlrp3) inflammasome, leading to IL-1β release in murine and human macrophages and human keratinocytes. Moreover, comparison of IL-1β production in response to exposure to various engineered NPs showed that high concentration (500 µg/mL) of smaller anatase and larger rutile TiO_2_ particles induced high production of IL-1β [31]. Another study showed that TiO_2_ nanobelts, but not P25 or anatase TiO_2_, induced IL-1β in THP-1 cells [32]. Yazdi *et al.* [30] also demonstrated that chemical ROS scavenger diminished IL-1β secretion triggered by TiO_2_ NPs in THP-1 cells, suggesting that ROS production induced inflammatory cytokine production after exposure to TiO_2_ NPs. On the other hands, other previous reports showed that ROS was not essential for IL-1β production via the Nlrp3 inflammasome [33,34]. In the present study, the production of IL-1β was significantly increased in THP-1 macrophages after exposure to 50 µg/mL of anatase TiO_2_ NPs. Since the level of ROS was also most elevated in THP-1 macrophages exposed to 50 µg/mL of anatase TiO_2_ NPs compared with other particles, it seems that anatase TiO_2_ nanoparticles induce inflammatory responses through accumulation of ROS in THP-1 macrophages. However, ROS might be not necessarily the main contributing factor of particles-induced IL-1β production in THP-1 macrophages because ROS level was increased after exposure to all TiO_2_ particles.

Orally ingested NPs are uptaken by epithelial cells and M cells in Peyer’s patches through the process of endocytosis, invasion by over-adsorption in cell gaps, and/or intrusion by passing through the tight junctions between cells [35]. Fine and ultrafine particles are potent adjuvants in antigen-mediated immune responses and are increasingly associated with inflammatory bowel diseases, such as Crohn’s disease [36]. ZnO NPs have been shown to induce cytotoxicity associated with overproduction of ROS in Caco-2 cells [37,38]. ZnO NPs has also been reported to induce inflammatory responses and increase the release of IL-8 in Caco-2 cells [37,39]. The present study found IL-8 over-expression in Caco-2 cells exposed to anatase TiO_2_ NPs for 6 h. Interestingly, a previous similar study showed that exposure to 10 µg/mL of P25 TiO_2_ NPs for 24 h led to increased IL-8 production in Caco-2 cells [40]. On the other hand, De Angelis *et al.* [39] demonstrated the induction of IL-8 production after 6-h exposure to ZnO NPs, but not anatase TiO_2_ NPs. The smaller mean hydrodynamic diameter of anatase TiO_2_ NPs estimated in the present study, relative to that of the above study [40] could perhaps explain the differences between the two studies. In the present study, there was no correlation between the induction of IL-8 production and ROS production in Caco-2 cells after TiO_2_ particles exposure. As shown in a previous report [41], oxidative stress induced by various NPs is an early event as the initial cellular response and ROS might not play a role in the impairment of inflammation-related pathway. TiO_2_ NPs can induce nuclear factor (NF)-κB activity by subsequent degradation of inhibitor (I)κ-B in airway epithelial and endothelial cells [42,43]. The presence of a binding site for NF-κB in the promoter region of IL-8 and enhanced IL-8 transcription following NF-κB binding, suggests that IL-8 expression could be up-regulated through NF-κB activation following exposure to anatase TiO_2_ NPs.

The present study indicated that anatase TiO_2_ NPs induced inflammatory responses compared with other TiO_2_ particles. However, Zijno *et al.* [44] recently compared the genotoxicity of TiO_2_ and ZnO NPs and demonstrated that only ZnO NPs were genotoxic, including destruction of micronuclei and DNA damage, although both NPs produced ROS in Caco-2 cells. Moreover, native TiO_2_ NPs and pretreated TiO_2_ NPs with the digestion simulation fluid or bovine serum albumin did not show significant toxicity in both Caco-2 cells and Caco-2 monolayers [45]. Janer *et al.* [46] suggested that the Caco-2 monolayer system is likely to underestimate the effects of oral absorption of NPs due to the fact that NPs were observed in Peyer Patch cells in the oral absorption study. The development of safe and effective NPs is important for advancement of technology and for healthy lives. There is no doubt a need to elucidate the effects and mechanisms of TiO_2_ NPs in the intestine using co-culture models, such as microfold (M) cells or intestinal epithelial cells ingested particles *in vivo*.

## 4. Experimental Section

### 4.1. TiO_2_ Particles Preparation and Characterization

The TiO_2_ particles used in the present study were A50 (anatase, primary diameter: 50 nm) (mkNano, Mississauga, ON, Canada), A100 (anatase, primary diameter: 100 nm) (mkNano), R50 (rutile, primary diameter: 50 nm) (mkNano), R250 (rutile, primary diameter: 250 nm) (mkNano), and P25 (80% anatases/20% rutile, primary diameter: 21 nm) (Degussa, Germany). We characterized previously P25 TiO_2_ NPs from the same lot by DLS as well as by transmission electron microscope (TEM, JEM-1011; JEOL, Tokyo, Japan), and then established a suitable protocol for the preparation of a suspension of TiO_2_ NPs [14]. NPs were suspended in serum-containing culture media and dispersed using a sonicator (model 450, Branson Sonifier, Danbury, CT, USA) set at 80% pulsed mode, 100 W, and 15 min. The hydrodynamic size of the particles in the medium was measured four times after 1 h on standing using DLS technology with zetasizer Nano-S (Malvern Instruments, Worcestershire, UK). The dispersion status was described by the intensity-weighted hydrodynamic average diameter (*z*-average) and PdI, which reflect the broadness of the size distribution (scale range from 0 to 1, with 0 being monodispersion and 1 being polydispersion). To investigate the electrophoretic mobility of the particles, ζ potential of the particles in each medium was measured three times with Photal LEZA-600 (Otsuka Electronics, Tokyo, Japan).

### 4.2. Cell Culture

Macrophages play a key role in the body’s defense to particles as well as in inflammatory-related health effects. Human monocytic leukemia cells cell line THP-1 (ATCC, TIB202, Manassas, VA, USA) were cultured in RPMI 1640 medium (Life Technologies, Carlsbad, CA, USA) supplemented with 10% (*v*/*v*) FBS, 100 units/mL penicillin, and 100 µg/mL streptomycin. THP-1 cells were differentiated to macrophages with 0.1 µg/mL phorbol 12-myristate 13-acetate (PMA; Sigma-Aldrich, St. Louis, MO, USA) for 72 h, before experimentation. The human colon colorectal adenocarcinoma cell line Caco-2 which are of intestinal epithelial origin, obtained from American Type Culture Collection (ATCC; HTB-37), were cultured in DMEM medium (Life Technologies) supplemented with 10% (*v*/*v*) FBS, 0.1 mM MEM NEAA, 100 units/mL penicillin, and 100 µg/mL streptomycin at 37 °C in 5% CO_2_.

### 4.3. Cell Viability Assay

THP-1 monocytes were seeded at 1.5 × 10^4^ cells/well on 96-well plates and differentiated to macrophages with PMA before the experiment as described above. Caco-2 cells were seeded overnight at 1.5 × 10^4^ cells per well on 96-well plates before the experiment. Particles were dispersed in each serum-containing cell culture medium at a final concentration ranging from 1 to 50 µg/mL. The previous studies demonstrated that TiO_2_ NPs induced a pronounced inflammatory response at the concentration of 10–200 µg/mL in *in vitro* models of gut epithelium [47]. Also, the concentration range corresponded to the dose used in our previous study [23]. Cell viability was determined after incubation with the dispersed TiO_2_ particles for 24 or 72 h, by MTS assay based on the CellTiter 96 AQueous One Solution (Promega, Madison, WI, USA), which measures mitochondrial function; the latter correlates with cell viability. The serum-containing cell culture medium was used during incubation with the particles. After the incubation, the cells were incubated with fresh medium (phenol red-free) containing MTS reagent for 1 h before measurements at an absorbance of 490 nm. The effect of particles on cell proliferation was expressed as percentage of inhibition of cell growth relative to the control.

### 4.4. Measurement of ROS Production

Cellular ROS production triggered by TiO_2_ particles was assayed by staining with 5-(and-6)-chloromethyl-2′,7′-dichlorodihydro fluorescein diacetate, acetyl ester (CM-H_2_DCFDA) (Life Technologies) followed by flow cytometry (FACS CantoII, BD Bioscience, Franklin Lakes, NJ, USA). Before the experiment, THP-1 monocytes were seeded at 3 × 10^5^ cells/well onto 24-well plates and allowed to differentiate into macrophages using PMA as described above. Caco-2 cells were seeded overnight at 3 × 10^5^ cells/well onto 24-well plates before the experiment. After exposure to TiO_2_ particles for 3 h, THP-1 macrophages and Caco-2 cells (3 × 10^5^ cells) were loaded with 5 µM CM-H_2_DCFDA for 30 min at 37 °C and analyzed by flow cytometry. Ten thousand cells per sample were acquired in histograms using FlowJo software (Flowjo, Ashland, OR, USA). Dead cells and debris were excluded by electronic gating using forward and side scatter measurements.

### 4.5. Measurement of IL-1β Production

Before the experiment, THP-1 monocytes were seeded at 1.5 × 10^4^ cells/well onto 96-well plates and differentiated to macrophages using PMA as described above. THP-1 macrophages were exposed to 25 or 50 µg/mL of the suspended particles for 24 h. The cell culture medium was collected and centrifuged at 10,000× *g* to remove cell debris and suspended TiO_2_ particles. The final supernatant was stored at −20 °C until cytokine analysis. The amount of IL-1β in the cell medium was measured using ELISA (Biolegend, San Diego, CA, USA) according to the protocol supplied by the manufacturer. Changes in color intensity were quantified by a plate reader (Bio-Rad Laboratories, Hercules, CA, USA).

### 4.6. Analysis of IL-8 Expression

Caco-2 cells (2 × 10^5^ cells) were seeded onto 12-well plates and exposed to 25 or 50 µg/mL of the suspended particles for 3 or 6 h. Total RNA from the cells was isolated by using ReliaPrep RNA cell miniprep system (Promega) using the protocol provided by the manufacturer. The concentration of total RNA was quantified by spectrophotometry (ND-1000; NanoDrop Technologies, Wilmington, DE, USA). RNA was reverse transcribed to single-strand cDNA using SuperScript III First-Strand Synthesis System for RT-PCR (Life Technologies). cDNA (*n* = 4 in each group) was subjected to quantitative PCR analysis with FastStart Universal Probe Master Mix (Roche, Basel, Switzerland) and primers for IL-8 using an ABI 7000 Real-Time PCR system (Life Technologies), as described previously [48]. The gene expression level was normalized to that of β-actin in the same cDNA.

### 4.7. Statistical Analysis

All parameters were expressed as mean ± standard deviation (SD). Differences between groups were analyzed by one-way analysis of variance (ANOVA) followed by Dunnett’s *post hoc* test. A *p* value less than 0.05 was considered statistically significant.

## 5. Conclusions

Since TiO_2_ nanoparticles are widely used in various fields, including the food industry, understanding their behavior and effects on the intestine is essential for risk assessment. In this study, we examined the effects of TiO_2_ particles of different crystal structures and sizes in Caco-2 cells and THP-1 monocyte-derived macrophages. Exposure to 50 µg/mL of anatase TiO_2_ nanoparticles increased the production of IL-1β in THP-1 macrophages and increased IL-8 expression in Caco-2 cells. These results indicate that anatase TiO_2_ nanoparticles, but not other TiO_2_ particles, seem to induce inflammatory response.

## Figures and Tables

**Table 1:** Characterization of TiO_2_ particles.

Particles	Primary Diameter (nm)	Medium	Hydrodynamic Size (nm)	PdI	ζ Potential (mV)
A50	50	RPMI1640 (10% FBS)	205.30 ± 4.88	0.319 ± 0.007	−11.86 ± 1.47
		DMEM (10% FBS)	227.78 ± 3.62	0.291 ± 0.012	−11.31 ± 0.39
A100	100	RPMI1640 (10% FBS)	262.10 ± 4.66	0.191 ± 0.026	−11.66 ± 1.38
		DMEM (10% FBS)	253.40 ± 4.11	0.171 ± 0.010	−11.84 ± 1.74
R50	50	RPMI1640 (10% FBS)	193.28 ± 1.37	0.120 ± 0.032	−13.15 ± 1.05
		DMEM (10% FBS)	194.20 ± 2.14	0.123 ± 0.011	−11.66 ± 0.64
R250	250	RPMI1640 (10% FBS)	439.13 ± 8.665	0.163 ± 0.010	−12.18 ± 0.59
		DMEM (10% FBS)	441.28 ± 6.65	0.155 ± 0.025	−11.75 ± 1.14
P25	21	RPMI1640 (10% FBS)	181.55 ± 1.10	0.153 ± 0.014	−13.10 ± 1.57
		DMEM (10% FBS)	193.85 ± 1.86	0.142 ± 0.008	−12.26 ± 1.11

Data are mean ± SD of three or four independent experiments. PdI: polydispersity index; RPMI: roswell park memorial institute; DMEM: dulbecco’s modified eagle’s medium; FBS: fetal bovine serum; TiO_2_: titanium dioxide.

## Abbreviations

NPs	nanoparticles
TiO_2_	titanium dioxide
DLS	dynamic light scattering
PdI	polydispersity index
ROS	reactive oxygen species
